# Bringing lived experience into research: good practices for public involvement in research

**DOI:** 10.1177/17579139221102229

**Published:** 2022-07-14

**Authors:** S Fowler Davis, C Woodward, B Greenfield, C Homer, K Williams, W Hameed, B Riley, D Roberts, G Bryan

**Affiliations:** Associate Professor, Advanced Wellbeing Research Centre (AWRC), Sheffield Hallam University (SHU), Sheffield, UK; Public Involvement in Research Group (PIRG) Co-ordinator, Advanced Wellbeing Research Centre (AWRC), Sheffield Hallam University (SHU), 2 Old Hall Road, Sheffield S9 3TU, UK; PIRG Member Advanced Wellbeing Research Centre (AWRC), Sheffield Hallam University (SHU), 2 Old Hall Road, Sheffield S9 3TU, UK; Early Career Researcher, Advanced Wellbeing Research Centre (AWRC), Sheffield Hallam University (SHU), Sheffield, UK; PIRG Member Advanced Wellbeing Research Centre (AWRC), Sheffield Hallam University (SHU), 2 Old Hall Road, Sheffield S9 3TU, UK; PIRG Member Advanced Wellbeing Research Centre (AWRC), Sheffield Hallam University (SHU), 2 Old Hall Road, Sheffield S9 3TU, UK; PIRG Member Advanced Wellbeing Research Centre (AWRC), Sheffield Hallam University (SHU), 2 Old Hall Road, Sheffield S9 3TU, Uk; PIRG Member Advanced Wellbeing Research Centre (AWRC), Sheffield Hallam University (SHU), 2 Old Hall Road, Sheffield S9 3TU, UK; PIRG Member Advanced Wellbeing Research Centre (AWRC), Sheffield Hallam University (SHU), 2 Old Hall Road, Sheffield S9 3TU, UK

Woodward et al. present this article as an example of good practice and reflection on the current development of a public involvement group.

The benefits of public involvement and engagement in research have been widely reviewed in health and care,^1,2^ with benefits that include the experience and worth gained by participants^3,4^ and the benefits to the research community.^5^ There is a growing awareness of the benefits of patient and public involvement (PPI) in research across disciplines, and acknowledgement of the need to address power inequities and a lack of diversity and inclusion.^2^ Innovative approaches to public involvement in multidisciplinary research are evolving and gaining more organisational commitment^6^ with researchers becoming better at accommodating public involvement and identifying engagement opportunities.

These considerations have been central to an organisational commitment at the Advanced Wellbeing Research Centre (AWRC), Sheffield Hallam University, where a new Public Involvement in Research Group (PIRG)^5^ was set up in July 2020. The vision to improve the health and wellbeing of the population was specifically focused on research and innovations that help people move, and the co-design of meaningful and high-quality research into physical activity. This article presents the process of setting up a PIRG within a research centre at a large academic institution. The article highlights the values of a PIRG and presents the areas identified by the members to develop the group and future impact. The article is co-authored by the public involvement group members and academic coordinators.

The key features of the PIRG member activity are bullet pointed below:

- 24 members of the public, two being co-chairs who attend internal governance meetings;
- A mixed approach to the review of research proposals: remote paper based and online live reviews;
- Quarterly members’ meetings, to provide updates on the research centres’ activity, progress on developments, feedback on previous reviewed bids;
- Selected co-applications, lay advisors and participation in research delivery;
- Periodic reviews of themes and programme and selected (optional) engagement in surveys, wider engagement with other PPI activity and business proposals.

Our aim was to recruit a varied individual contribution and to sustain membership of the PIRG to engage fully with the new research centre. Higher education innovation funding (HEIF) was used to enable a funded partnership with Healthwatch UK^7^ and a planned recruitment across the city. The collaboration enabled us to access a wide range of communities, voluntary sector and statutory organisations, to promote the opportunity to be involved with the AWRC. The initial recruitment sought to reflect local populations and communities while remaining ‘generic’ in research focus, that is, inclusive of the widest range of values and opinions associated with wellbeing and long-term conditions, prevention and management. By working in partnership and addressing the equality and diversity constraints that are known to limit the range of perspectives for research,^8^ the partnership sought to build on a commitment to be inclusive in hearing from different groups and enabling participation in research.^9^

A coordinator was recruited from the AWRC researcher community and the AWRC Board^i^ recognised how the PIRG would amplify a user perspective in the AWRC with this statement included in the terms of reference:

*Public involvement is seen as a valuable and essential part of the way research is prioritised, designed, run and shared. It is seen to improve the quality and running of research projects, inform the exchange of knowledge between researchers and practice, and drive the translation of research and its positive impact on people beyond the academic community.*

The operational processes have been set up with specific projects to ensure the security of member’s personal data, a standardised method for payment and an induction programme that enables experienced and less experienced members to contribute fully. These processes are important and by clarifying the support for members, the potential imbalances of power are addressed and access to support is made clear.

The impact of the PIRG has recently been evaluated^10^ through a series of online and face-to-face events and engaging in ‘learning conversations’.^11^ These were designed to enable open communication, make people feel comfortable to speak and to encourage participation in planning, with a view to planning further recruitment. The outcomes of these sessions help the organisation to deepen the engagement and involvement, and learning has been grouped into ‘themes of concern’.

1. *Creating a space for safe involvement*: The membership, now 24 individuals, has remained consistent since the start, and members have valued the structure and the administrative support that enables their voluntary contribution. The comments included, it’s ‘well set up, enough members and good staff support’. There is a respect for different ways that individuals participate. ‘I don’t always contribute but when I do I feel that I am being listened to which is important’. Some members found that there was ‘way too much talking to each other and administration in the meetings and far too little of what I am interested in’ and so a couple of members have elected to just review research proposals and not to participate in meetings. Others see themselves as ‘team players with researchers’ and actively respond. It has been important to the group to check the level of confidentiality required for individual projects, which is regularly communicated by the researcher and transferred via the coordinator.
2. *Reward and purpose*: Both PIRG members and researchers have commented on the value of participation: ‘From my own point of view, I am finding my involvement both thought-provoking and rewarding, and believe we are helping the AWRC to be effective as a national research centre, but also as a focus for the wellbeing of the surrounding community’. Members have attended AWRC Board meetings and have absorbed the mission to engage in applied research as a core purpose of the AWRC: ‘I like to think the PIRG reviewer can help the translational process, taking good ideas from pure research to an applied solution that can be deployed in the real world’. Many researchers are unfamiliar with sharing their research ideas with lay members but have also responded well, commenting on the feedback they receive: ‘ensuring we have the public voice to check, challenge and improve the research we undertake is so important. I look forward to taking the next steps to PI and including PIRG members as co-applicants on our bids’. PIRG members often make constructive suggestions about patient facing documents and try to ensure good use of plain English. Another frequent area of scrutiny is how patient data are to be safeguarded and kept anonymised. Both types of involvement reinforce the need for researchers to follow best practice.
3. *Equality, diversity and inclusion*: As a core value of the PIRG, the lived experience is always the rationale and often the motivation to become and remain a member of the group. Members review draft research proposals, responding to researcher’s ideas from a personal perspective. The range and demographic of the membership are diverse in age, gender and cultural perspective, but there is an ongoing desire to extend the breadth of experience.^11^ ‘One of the challenges is having good representation within the group of the community around us, the people we aim to help with our research, the real-life experiences to challenge the academics thinking’. Current membership is supported by some members who represent themselves and others from their networks and communities and this has enabled a wide range of opinion and diversity of views. Examples include ‘walking groups’, ethnically diverse third-sector organisations and underrepresented communities, that is, young carer services. A key advantage of this approach is that working-age people are involved alongside those who are retired and not in formal employment. Several PIRG members are also expert patients and so have useful insight into how health services are currently provided and, for example, how patient organisations like the British Heart Foundation (BHF) encourage cardiology patients to exercise safely, even during periods of lockdown.

Experiences of exercise programmes vary between members, from positive applications in accelerated recovery programmes following surgery to less positive experiences of graded exercise therapy (GET). These perspectives are helpfully shared with researchers to consider when shaping their studies. The same is true of diet regimes; some members understand through their own experience how to incentivise people to lose weight in practical ways and others have strong views about how people from different cultural backgrounds may need tailored approaches to managing body shape and healthier weight through exercise.

*Actions from the discussion* have been used to formulate a range of improvement activity that will be undertaken with the group members and across the AWRC. The most pressing is the continued active involvement of all researchers, particularly those in disciplines that are unfamiliar with exposing their research ideas to feedback from individuals and communities. We are encouraging ‘early enthusiasts’ to share their experience: ‘We received lots of detailed feedback. I was grateful to see how much time and effort the reviewers had put into assessing our proposal. I would definitely bring future projects to PIRG for review’.

The other ongoing commitment is to build continuous improvement in representation and diversity. Members are already trying to ensure that those who have less ability to engage with research are invited to participate. Strengthening public involvement and engagement^12^ is often associated with training and development, particularly for those who are new to research and to framing feedback. Reporting the impact of PPI in research should include how people with highly embedded and relevant experience are identified and supported.^13^ The goal of the formal PPI group is to enable detailed insights to inform the research and provide researchers a real opportunity to learn through lived experience of different populations, reporting this alongside key research findings.^14^

By working with a subset of the group, a development plan for 2022 is now in place to

1. Enhance the offer to academics by way of sharing learning from previous reviews, increasing access to and awareness of the group to enhance their research, increasing diversity within the group to bring a wider range of lived experience.
2. Enhance the experience for the members of public by way of improving communication channels within the group, informing them about wider activity of the Research Centre and College which it sits, supporting with training for those new to PPI, ensuring the group and review methods are accessible to all.

## Summary

Innovation in public involvement is based on continuous improvements to processes and systems that enable a sustained infrastructure that allows members to offer feedback to researchers about their research. Engagement requires continuous learning and development with the existing group and with the researchers undertaking complex multidisciplinary studies. Our PIRG evaluated activity after 18 months of operating, and this article reports on the value of developing a safe infrastructure to support, develop and grow collaborations, and methods of enabling all PIRG members to focus on the impact of the studies in improved health and wellbeing outcomes.

Due to positive experience of public involvement shaping research projects for the better, the AWRC is not only seeking to make the work of the PIRG more widely accessible to researchers but also to ensure PIRG continues to engage with relevant, underrepresented health service user groups.

## Notes

i. The Board of the Advanced Wellbeing Research Centre (AWRC) is the senior group responsible for the governance of the research.
